# Phylogenetic Analyses of True Ladybirds (Coleoptera: Coccinellidae: Coccinellini) Reveal Directionality in Diet Evolution and Support a Boreotropical Origin of the Tribe

**DOI:** 10.1002/ece3.73077

**Published:** 2026-03-26

**Authors:** Romain Nattier, Noémie M.‐C. Hévin, Pascaline Chifflet‐Belle, Jiajia Dong, Hermes E. Escalona, Julien M. Haran, Jean‐Claude Streito, Wioletta Tomaszewska, Adam Slipiński, Andreas Zwick, Gael J. Kergoat

**Affiliations:** ^1^ CNRS, SU, EPHE‐PSL, UA, Institut de Systématique, Evolution, Biodiversité (ISYEB) Muséum National d'Histoire Naturelle Paris France; ^2^ CNRS, IRD, EPHE, Institut des Sciences de l’Evolution de Montpellier Université de Montpellier Montpellier France; ^3^ Jiangsu Key Laboratory of Brain Disease and Bioinformation, Research Center for Biochemistry and Molecular Biology, School of Life Sciences Xuzhou Medical University Xuzhou China; ^4^ Australian National Insect Collection, CSIRO Canberra Australian Capital Territory Australia; ^5^ CBGP, CIRAD, INRAE, IRD, Institut Agro University of Montpellier Montpellier France; ^6^ Museum and Institute of Zoology Polish Academy of Sciences Warsaw Poland

**Keywords:** Bayesian stochastic mapping, biogeography, boreotropics, Coccinellidae, diet evolution, host shifts

## Abstract

True ladybirds (Coleoptera: Coccinellidae: Coccinellini) are an iconic and beloved group of insects comprising *c*. 1000 species. Contrary to common belief, true ladybirds are not restricted to temperate areas in the Northern hemisphere and can be found on every continent (with the exception of Antarctica). Moreover, although they are commonly viewed as aphid specialists, they actually have a wider range of diets, with some species being, for example, mildew fungus specialists. To better understand their biogeographic history and diet evolution, we generate the current largest dated molecular phylogeny of Coccinellini, based on a multi‐marker molecular dataset (three mitochondrial and eight nuclear gene fragments) for 206 species representing 57 genera, plus 44 outgroups. The resulting phylogenetic hypothesis clarifies the relationships between main clades and genera while providing a more comprehensive framework to draw inferences on the evolution of the tribe. Our historical biogeography analyses reveal a dynamic but highly structured pattern for the tribe, consistent with the hypothesis of a boreotropical origin during the Paleocene. From there, numerous lineages spread southward, with multiple independent colonizations of the Afrotropical, Australasian, and Neotropical regions. Bayesian stochastic mapping analyses also highlight interesting patterns, such as the pivotal role of the East Palearctic region and the importance of Beringian land bridges in the dispersal of lineages toward the Nearctic. Ancestral character state estimation analyses recover a structured pattern, with an ancestral association on aphids often followed by a broadening of the host repertoire and secondary shifts onto different food sources. These analyses also support the existence of a directionality in diet evolution, where expansion of the host repertoire constitutes a prerequisite for shifting to a novel food resource; interestingly, the only marked exception to this pattern is the single shift from aphids to mildew fungi leading to the diversification of a specific mycetophagous lineage.

## Graphical abstract

A newly generated large‐scale molecular phylogeny of true ladybirds (Coccinellini) clarifies their evolutionary relationships and supports a boreotropical origin in the Paleocene, with multiple independent southward dispersals. Ancestral diet reconstructions suggest an initial specialization on aphids, followed by expansions in host range and occasional shifts to alternative food sources, with one notable exception involving a direct transition to mildew fungi.

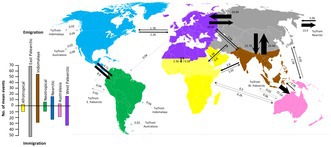

## 1 Introduction

Ladybird (or ladybug) beetles (Coccinellidae) are among the most iconic of insects. Renowned for their generally bright, striking colors and easily recognizable rounded shape, they are widely associated with good luck and are often regarded as symbols of protection and prosperity (Majerus 2016) and hold a special place in popular culture. However, despite their iconic status, largely attributed to a few well‐known species such as the seven‐spotted ladybird *Coccinella septempunctata* L., ladybirds present a remarkable diversity (360 genera and over 6000 species). They also exhibit a wide range of sizes (from 0.8 to 13 mm), and a high diversity of trophic relationships, with most species being predators of sternorrhynchan Hemiptera, alongside several phytophagous and mycetophagous lineages (Evans 2009; Giorgi et al. 2009; Sutherland and Parrella 2009; Ślipiński and Tomaszewska 2010). Importantly, both larvae and adults generally have a similar diet (Majerus 1994; Hodek and Honēk 1996; Vandenberg 2002; Giorgi et al. 2009). Several species have been intentionally introduced for biological control, such as the vedalia beetle *Novius cardinalis* (Mulsant), whose introduction from Australia to California marked the beginning of classical biological control (Caltagirone and Doutt 1989). It should be noted that one species initially used for biological control, the harlequin ladybird *Harmonia axyridis* (Pallas), is now invasive worldwide (Roy et al. 2016), causing decline of local ladybird species while also impacting food production and human health (De Groot and Haelewaters 2022). Some ladybird species are also crop pests, such as the 28‐spot ladybird *Henosepilachna vigintioctopunctata* (Fabricius), which is a major pest of solanaceous crops (Wang et al. 2017).

**FIGURE 1:**
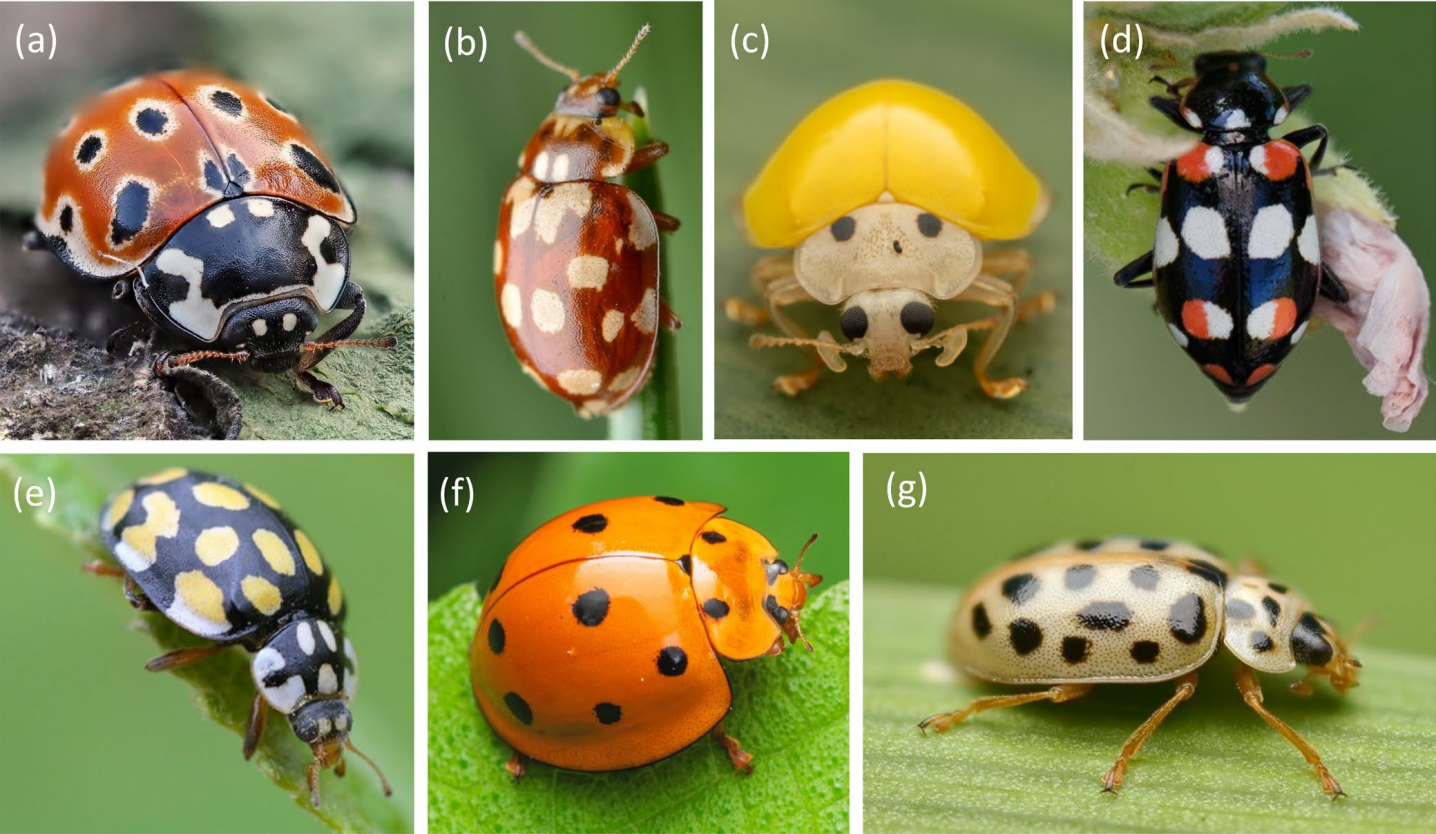
Adult representatives of major lineages of Coccinellini: (a) *Anatis ocellata* (L.) (picture by G. San Martin); (b) *Myrrha octodecimguttata* (L.) (picture by G. San Martin); (c) *Illeis confusa* Timberlake (picture by B. Heath); (d) *Eriopis connexa* (Germar) (picture by G. Brachetti); (e) *Sospita vigintiguttata* (L.) (picture by G. San Martin); (f) *Megalocaria dilatata* (Fabricius) (picture by A. Hardacre); (g) *Anisosticta novemdecimpunctata* (L.) (picture by G. San Martin).

Within the family Coccinellidae, the tribe Coccinellini (also known as “true ladybirds”) is the second most species‐rich (over 1000 species in 91 genera; see Escalona et al. 2017 and González et al. 2022; Figure 1). Our knowledge of the tribe's evolutionary history first benefited from several molecular studies relying on a rather limited number (between 23 and 38) of Coccinellini species (Aruggoda et al. 2010; Magro et al. 2010; Robertson et al. 2015; Escalona et al. 2017). Although these studies helped to better circumvent the boundaries of the tribe, their phylogenetic resolution and robustness were somewhat limited, due to a combination of sparse sampling and low numbers of molecular markers. A significant step forward in our understanding of the evolution and systematics of the tribe later came from two studies (Nattier et al. 2021; Tomaszewska et al. 2021), which sampled more species and genetic markers. These studies focused primarily on clarifying the higher systematics of the tribe and the boundaries of major clades while also investigating the timing of its diversification; both studies yielded fairly congruent results, recovering similar clades and a comparable timeframe for the tribe, with a Late Cretaceous origin. Tomaszewska et al. (2021) also investigated the evolution of several morphological characters, highlighting that characters frequently used for genus delineation were highly homoplastic.

The majority of Coccinellini species are found in temperate regions (Iablokoff‐Khnzorian 1982; Gordon 1985; Ślipiński and Tomaszewska 2010; Hounkpati et al. 2020; Ślipiński et al. 2020), contrary to most coccinellid tribes that display a latitudinal diversity gradient pattern, characterized by greater diversity in tropical and subtropical regions (Willig et al. 2003). While an origin of Coccinellini in the East Palearctic has been postulated (Iablokoff‐Khnzorian 1982), it has never been properly tested, and the absence of unequivocal Coccinellini fossils (Szawaryn and Szwedo 2018) are a major impediment. Inferring their colonization routes between major continental blocks is also of particular interest in the general context of Northern and Southern hemisphere biogeography (Sanmartín et al. 2001; Sanmartín and Ronquist 2004). It is especially the case for transcontinental dispersion between the Nearctic and the Palearctic, where major alternative routes have been identified, either between the East Palearctic and West Nearctic (Beringian land bridges I, II, III; Sanmartín et al. 2001; Wen et al. 2016) or between the West Palearctic and the East Nearctic (Thulean and De Geer routes; Sanmartín et al. 2001; Brikiatis 2014). The Early Oligocene drying of the Turgai Strait is another major event that supposedly favored dispersal between the West and the East Palearctic (Rögl 1999; Hou et al. 2011; Čerňanský and Augé 2019). In the Southern hemisphere, studies on mammals showed that dispersals between the Afrotropics and the Palearctic were first facilitated during the Paleocene by the Alboran/Apulia route in the western Mediterranean, then in the late Eocene by the Iranian route in the eastern Mediterranean (see the review of Gheerbrant and Rage 2006). More extensive biotic exchanges were promoted later (e.g., Toussaint et al. 2019) by the collision of the Arabian plate with Eurasia from *c*. 27 Ma (McQuarrie and van Hinsbergen 2013) and the closure of the Tethys Sea during 24–21 Ma (Torftstein and Steinberg 2020). Dispersal between Australasia and the Indomalaya was favored by a succession of geological events, starting with the collision of the Australian plate with Southeast Asia *c*. 23 Ma (Hall 2011), which led to the formation of several archipelagos that acted as potential stepping stones in a context where sea level started falling in the Early Miocene (Hall 2012). Subsequently, landmasses in the Indo‐Australian Archipelago (IAA) began to emerge between the Late Miocene and the end of the Pliocene (10–2.58 Ma), increasing biotic exchange between Southeast Asia and Australia (Lohman et al. 2011). In the Western hemisphere, biotic exchanges between the Nearctic and Neotropics were also promoted by the putative GAARlandia landbridge at the Eocene–Oligocene boundary (Iturralde‐Vinent and MacPhee 1999; but see Ali and Hedges 2021), the Panama Island Arc (15–13 Ma; Montes et al. 2015) and the closing of the Panama Isthmus sometimes between the Late Miocene and the Pliocene (O'Dea et al. 2016; Molnar 2017).

The biogeographic history and extant species distribution of Coccinellini has also been partially impacted by their feeding preferences. Indeed, the tribe is best known for their feeding specialization on aphids (Hemiptera: Aphididae), a group that also displays higher levels of diversity in the temperate environments of the Northern hemisphere (Dixon et al. 1987; Blackman and Eastop 2000; von Dohlen and Teulon 2003). However, Coccinellini are not only aphid specialists; several lineages can supplement their diet and can also prey on other insect groups (heteropterans, psyllids, beetle or moth larvae), or specialize entirely on either other insect groups (Evans 2009) or on mildew fungi (Sutherland and Parrella 2009). Two studies (Escalona et al. 2017; Nattier et al. 2021) investigated the evolution of feeding habits, recovering an ancestral association with aphids and a relatively dynamic pattern of diet evolution; however, both studies investigated a rather limited number of Coccinellini species (36 and 75, respectively) and did not implement alternative models allowing for distinct and non‐symmetrical rates of evolution.

To improve our understanding of the evolutionary history of Coccinellini, we infer a comprehensive phylogenetic framework (206 Coccinellini species representing 57 genera, plus 44 outgroups) based on 11 gene fragments. Using this new phylogenetic framework, we aim to: (i) provide a more comprehensive view of the evolutionary relationships within the tribe Coccinellini, (ii) reconstruct for the first time the biogeographic history of the group by leveraging time‐stratified parametric biogeographic models, and (iii) infer the evolution of diet with a focus on exploring the transition rates between distinct feeding regimes.

## 2 Materials and Methods

### 2.1 Taxon and Molecular Sampling

For this study, we aimed at achieving the most comprehensive multi‐marker molecular dataset possible for the tribe Coccinellini, in order to conduct in‐depth macroevolutionary analyses.

To do so, the taxon and molecular sampling for this study was built on the sampling achieved for the Coccinellini tribe by two recent studies that present the advantage of having 46 Coccinellini species in common as well as partially overlapping molecular datasets: (i) the study of Nattier et al. (2021), which includes 88 Coccinellini species, with a molecular dataset consisting of three mitochondrial (mtDNA) gene fragments (ribosomal 12S RNA “12S”, ribosomal 16S RNA “16S” and cytochrome *c* oxidase subunit I “COI”) and five nuclear gene fragments (18S ribosomal DNA “18S”, 28S ribosomal DNA “28S”, the “CADXM” fragment of carbamoyl‐phosphate synthetase, histone subunit 3 “H3”, and Wingless “Wg”), and (ii) the study of Tomaszewska et al. (2021), which includes 162 Coccinellini species (46 previously sampled in the study of Nattier et al. 2021), with a molecular dataset consisting of a COI fragment and five nuclear (nDNA) gene fragments (arginine methyltransferase “Art1”, the “CADMC” and “CADXM” fragments of carbamoyl‐phosphate synthetase, DNA topoisomerase I “Top1” and Wg); hence three markers (COI, CADXM and Wg) were sequenced in both studies.

To increase the level of molecular data overlap and densify the taxon sampling, we generated new sequences for 78 Coccinellini species (see Table S1 for the primer list). In all, this resulted in the most comprehensive sampling of the tribe ever achieved, with 206 Coccinellini species representing 57 distinct genera (out of 91). In addition, we included 44 species as outgroups (42 Coccinellidae species representing the three subfamilies and 13 tribes, plus one representative of Cerylonidae and Gyrinidae). All newly generated sequences were deposited in GenBank (see Tables S2 and S8 for all accession numbers and the complete list of species, outgroups included). All gene fragments were individually aligned using MAFFT v.7 (Katoh et al. 2019) with default settings, then merged using Mesquite v.3.81 (Maddison and Maddison 2023). Finally, the quality of alignment was checked manually (specifically the poorly aligned regions as well as the presence of stop codons), resulting in a molecular dataset of 250 species (including 206 Coccinellini species), and 11 gene fragments totalling a length of 10,878 nucleotides for the multiple segment alignment.

### 2.2 Molecular Phylogenetics

Molecular phylogenetic analyses were carried out under maximum likelihood (ML) using IQ‐TREE v2.2.2.7 (Minh et al. 2020). The concatenated dataset was split into 29 partitions a priori, with three partitions (one per codon position) defined for each of the coding gene fragments (Art1, CADMC, CADXM, COI, H3, Top1, Wg) and one partition for each of the non‐coding gene fragments (12S, 16S, 18S, 28S); for CADMC, due to the presence of an intron, seven partition were used. The Bayesian Information Criterion implemented in IQ‐TREE through ModelFinder (Kalyaanamoorthy et al. 2017) was used to select best‐fit substitution models and partition schemes (see Table S3). On the basis of the results of the study of Zhang et al. (2018), a reduced Coleoptera phylogenomic backbone encompassing four species ( *Anisosticta bitriangularis* (Say), *Coccidophilus* sp., *Delphastus* sp. and *Monocoryna* sp.) was enforced (‐*g* option), and the gyrinid *Macrogyrus oblongus* (Boisduval) was used to root the tree. The best‐fit ML tree was obtained by running a series of ML analyses with a range of distinct values of perturbation strength (0.0, 0.2, 0.4, 0.6, 0.8 and 1.0; *‐pers 0.x* option). For each perturbation strength value, we carried out 20 independent heuristic searches with the following settings: hill‐climbing nearest neighbor interchange (NNI) search (*‐allnni* option), partition‐resampling strategy (*‐sampling GENE* option), best partition scheme allowing the merging of partitions (*‐m MFP + MERGE* option). The ML tree with the best log‐likelihood score also had the best AICc and AICcω (see Table S4), and was selected as the reference tree for further analyses. Clade support for all analyses was assessed using 1000 replicates for both SH‐like approximate likelihood ratio tests (SH‐aLRT; Guindon et al. 2010) and ultrafast bootstraps (uBV; Minh et al. 2013). According to the authors' recommendations, branches with SH‐aLRT values ≥ 80 and uBV values ≥ 95% were considered highly supported (thresholds based on Guindon et al. 2010 and Minh et al. 2013 for SH‐aLRT and uBV, respectively; see also Anisimova et al. 2011 for a detailed discussion on branch support metrics).

### 2.3 Dating Analyses

Divergence times were estimated using Bayesian relaxed clocks as implemented in BEAST v1.10.4 (Suchard et al. 2018). For this study, we implemented a node‐dating approach relying on secondary calibrations derived from the phylogenomic study of Che et al. (2021). The following calibration points were enforced using uniform distributions: (i) most recent common ancestor (MRCA) of Coccinellidae (minimum age of 127.5 million years ago (Ma) and maximum age of 158.1 Ma); (ii) MRCA of Coccinellinae (minimum age of 92.4 Ma and maximum age of 120.5 Ma); (iii) MRCA Microweiseinae (minimum age of 62.7 Ma and maximum age of 104.3 Ma). The best‐scoring tree from the IQ‐TREE analysis was used, with two distinct uncorrelated lognormal clocks (one for the three mtDNA genes and one for the eight nDNA genes), and the tree model set to a birth‐death speciation process (Gernhard 2008). BEAST analyses consisted of four distinct runs of 50 million generations of Markov chain Monte Carlo (MCMC) with parameters and trees sampled every 2500 generations. A 25% burn‐in was further applied, and the post burn‐in trees and log files from the four runs were combined using LogCombiner v1.10.4, which is part of the BEAST software package. The maximum credibility tree, median ages and their 95% HPD were further produced using TreeAnnotator v1.10.4. Convergence of runs was evaluated graphically and by looking at the effective sample size (ESS) of relevant parameters under Tracer v1.7.2 (Rambaut et al. 2018), using the recommended threshold of 200 for relevant parameters.

### 2.4 Historical Biogeography

Specimen distribution data for each species were gathered from multiple sources that are listed in Table S5. Historical biogeography analyses were carried out using parametric methods (Ree and Sanmartín 2009) with the *R* package BioGeoBears (Matzke 2013, 2014). We implemented the following three models: (i) dispersal‐vicariance analysis (DIVA; Ronquist 1997), (ii) Dispersal–Extinction–Cladogenesis (DEC; Ree and Smith 2008), and (iii) Bayesian inference of historical biogeography for discrete area (BAYAREA; Landis et al. 2013). The area categorization was based on seven standard biogeographical regions: (i) Afrotropics [A], (ii) East Palearctic [E], (iii) Indomalaya [I], (iv) Neotropical [N], (v) Nearctic [R], (vi) Australasian [U], and (vii) West Palearctic [W]. To reflect extant Coccinellini distribution patterns, a maximum number of three areas was allowed.

We then defined a time‐stratified model with five time slices (TS), corresponding to the Paleocene (TS5; 65–56 Ma), the Eocene (TS4; 56–33.9 Ma), the Oligocene (TS3; 33.9–23.03 Ma), the Miocene (TS2; 23.03–5.333 Ma), and the Pliocene to the end of the Pleistocene (TS1; 5.333–0.1 Ma). Following Kawahara et al. (2023), we added a last time slice (TS0; 0.1–0 Ma) where all combinations of areas were allowed so that the adjacency matrix was less strict. To account for change of connectivity through time between the biogeographical regions, for each time slice we implemented a matrix of scaling factors for dispersal rates (DR) between areas; areas that were either non‐adjacent or not connected by a potential landbridge or island‐hopping archipelago were excluded. Non‐adjacent areas or areas with no potential connection were systematically assigned a DR of 0.01; higher DR values ranging from 0.1 to 1.0 were set for the other combinations of areas. These matrices largely follow the dispersal matrices defined for the same areas by Kawahara et al. (2023), with slight modifications (Table S6). Analyses were further implemented with and without the founder‐event speciation parameter (+j) *sensu* Matzke (2014). As a guide tree, we used the dated phylogeny estimated with BEAST after removing all species not belonging to the Coccinellini tribe using Mesquite (“prune clade” tool). The best‐fitting model was selected based on the Akaike information criterion corrected for sample size (AICcω).

In addition, Bayesian stochastic mapping (BSM) analyses (Dupin et al. 2017) were performed under BioGeoBears with the best‐fitting model previously selected by the AICcω to estimate the number and type of biogeographic events with 50 stochastic replicates on 50 post‐burnin trees randomly sampled from the BEAST analysis.

### 2.5 Ancestral Character State Estimations

Information on the diet of species was gathered from multiple sources that are listed in Table S7. We managed to find information on diet for 119 species out of the 206 Coccinellini species in our dataset. Known feeding regimes were categorized using one character state with four states being defined: (i) aphidophagous, (ii) feeding on other insect groups than aphids (heteropterans, psyllids, beetle or moth larvae), (iii) mixed diet (both aphids and another food source such as other insect groups or pollen), and (iv) mycetophagous (feeding on mildew fungi). As a guide tree, we used the dated phylogeny estimated with BEAST; this tree was modified using *the drop.tip* function from *R* package ape 5.7‐1 (Paradis and Schliep 2019) by removing all species not belonging to the Coccinellini tribe and the Coccinellini species for which we had no reliable information on their diet. Ancestral character state estimation (ASE) analyses were carried out with the *R* package Phytools (Revell 2012) by fitting and comparing different rate transition matrices of the Markov k state (Mk) model for discrete characters. We compared the performance of the following three Mk models: (i) the equal‐rates model (ER), in which a single parameter governs all transition rates; (ii) a symmetric model (SYM), in which forward and reverse transitions share the same parameter; and (iii) an all‐rates‐different model (ARD), in which each rate is a unique parameter. These three models were fitted on the pruned tree with the *fitMk* function, and the best‐fitting model was selected based on Akaike information criterion weights (AICω). Finally, the ASE analysis with the best‐fitting model was conducted using stochastic character mapping as implemented with the *make.simmap* function, with 1000 simulations.

## 3 Results

### 3.1 Molecular Phylogenetics

**FIGURE 2:**
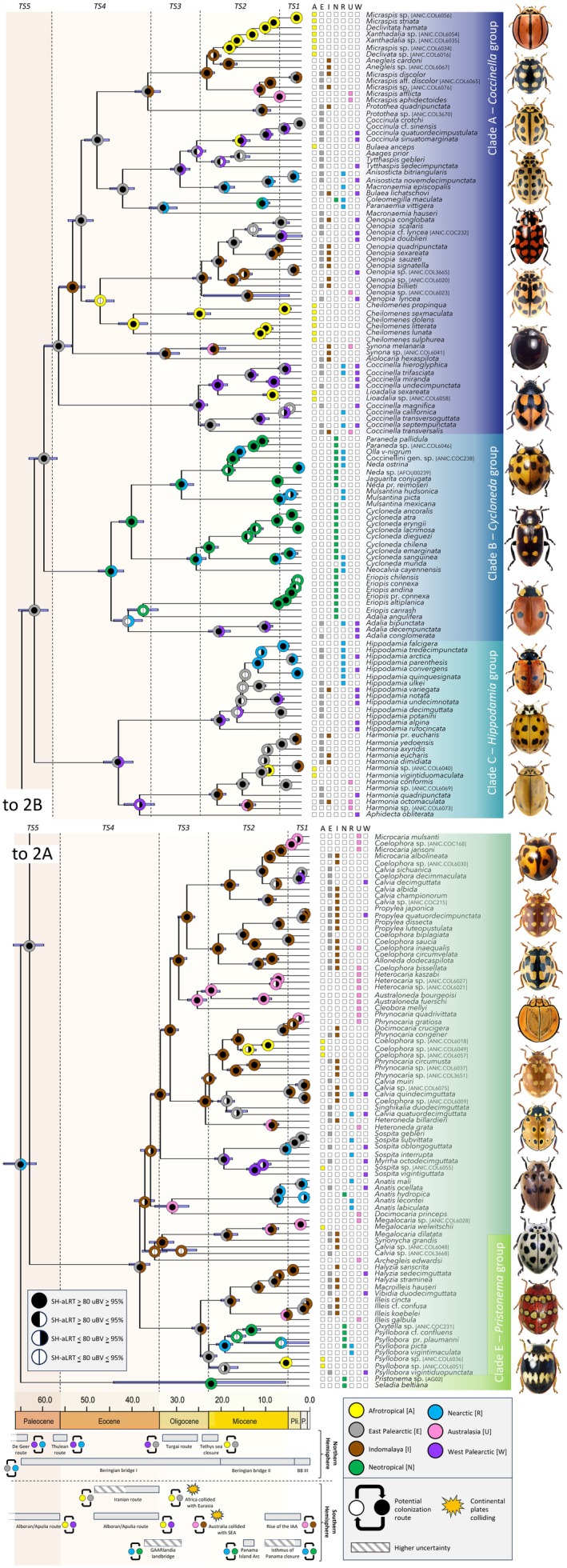
Dated phylogeny and historical biogeography of Coccinellini. Support values from ML phylogenetic analyses (SH‐aLRT and uBV) are represented by circles on nodes, and 95% HPD of ages from BEAST analyses by blue bars. The most likely ancestral states inferred from the best‐fitting model (DEC + j) are presented. The five time slices (TS1 to TS5) are represented in the background using colored bands. On the bottom, the timelines of several putative colonization routes and geological events are presented. Images of representatives from the tribe Coccinellini are presented on the right (pictures by G. González and U. Schmidt). From top to bottom: *Declivitata olivieri* (Gerstäcker), *Coccinula quattuordecimpustulata* (L.), *Tytthaspis sedecimpunctata* (L.), *Anisosticta novemdecimpunctata* (L.), *Coleomegilla quadrifasciata* (Schöenherr), *Oenopia conglobata* (L.), *Cheilomenes sexmaculata* (Fabricius), *Coccinella hieroglyphica* L., *Neda amandi* Mulsant, *Eriopis altiplanica* Gonzalez, *Adalia bipunctata* (L.), *Hippodamia variegata* (Goeze, 1777), *Harmonia axyridis* (Pallas), *Aphidecta obliterata* (L.), *Coelophora inaequalis* (Fabricius), *Calvia quatuordecimguttata* (L.), *Australoneda bielawskii* Li, Ślipiński & Pang, *Myrrha octodecimguttata* (L.), *Anatis ocellata* (L.), *Oxytella subcordata* Weise, *Psyllobora kirschi* Mulsant, *Pristonema erotyloides* (Guérin‐Méneville), *Seladia bicincta* (Mulsant).

The best‐scoring ML tree (*L* = −251,003.225) was obtained with a perturbation strength value of 1.0 (see Table S4). The resulting tree (see Figure 2 for the Coccinellini clade and Figure S1 for the complete tree) supports the monophyly of Coccinellini with maximum support (SH‐aLRT of 100 and uBV of 100%). Within the tribe, five highly supported (SH‐aLRT ≥ 80/uBV ≥ 95%) clades can be found, including four (clades A–D) that were previously uncovered in the studies of Nattier et al. (2021) and Tomaszewska et al. (2021). Additionally, we identified a novel clade, designated here as Clade E (*Pristonema* group).

Clade A (*Coccinella* group) is a diverse clade with a worldwide distribution. Our sampling encompasses 20 genera, of which 13 (*Aaages* Barovsky, *Aiolocaria* Crotch, *Anegleis* Iablokoff‐Khnzorian, *Anisosticta* Chevrolat, *Cheilomenes* Chevrolat, *Coccinula* Dobzhansky, *Coleomegilla* Cockerell, *Lioadalia* Crotch, *Oenopia* Mulsant, *Paranaemia* Casey, *Protothea* Weise, and *Synona* Pope) are inferred as monophyletic with a high support (SH‐aLRT ≥ 80/uBV ≥ 95%). The remaining seven genera (*Bulaea* Mulsant, *Coccinella* L., *Declivitata* Fürsch, *Macronaemia* Casey, *Micraspis* Dejean, *Tytthaspis* Crotch, and *Xanthadalia* Crotch) are not monophyletic.

Our sampling for Clade B (*Cycloneda* group) consists of nine genera, primarily distributed in the Western hemisphere, except for a few species of *Adalia* Mulsant which are also found in the Eastern hemisphere. In this clade seven genera (*Cycloneda* Crotch, *Eriopis* Mulsant, *Jaguarita* González et al., *Mulsantina* Weise, *Neocalvia* Crotch, *Olla* Casey, and *Paraneda* Timberlake) are monophyletic with a high support (SH‐aLRT ≥ 80/uBV ≥ 95%), whereas two are non‐monophyletic (*Adalia* and *Neda* Mulsant). Regarding clade C (*Hippodamia* group), we sampled three genera (*Aphidecta* Weise, *Harmonia* Mulsant, and *Hippodamia* Dejean) that are monophyletic with a high support (SH‐aLRT ≥ 80/uBV ≥ 95%).

Sampled representatives of clade C are absent from the Neotropical region. Similar to clade A, clade D (*Synonycha* group) is a diverse clade with a worldwide distribution. Our sampling encompasses 24 genera, of which 17 (*Alloneda* Iablokoff‐Khnzorian, *Anatis* Mulsant, *Archegleis* Iablokoff‐Khnzorian, *Australoneda* Iablokoff‐Khnzorian, *Cleobora* Mulsant, *Halyzia* Mulsant, *Heterocaria* Timberlake, *Heteroneda* Crotch, *Illeis* Mulsant, *Macroilleis* Miyatake, *Megalocaria* Crotch, *Myrrha* Mulsant, *Oxytella* Weise, *Propylea* Mulsant, *Shinghikalia* Kapur, *Synonycha* Chevrolat, and *Vibidia* Mulsant) are monophyletic with a high support (SH‐aLRT ≥ 80/uBV ≥ 95%). The remaining seven genera (*Calvia* Mulsant, *Coelophora* Mulsant, *Docimocaria* Crotch, *Microcaria* Crotch, *Phrynocaria* Timberlake, *Psyllobora* Dejean, and *Sospita* Mulsant) are not monophyletic. Clade E (*Pristonema* group) is a highly supported (SH‐aLRT ≥ 80/uBV ≥ 95%) new group comprising *Pristonema* sp. and *Seladia beltiana* Gorham, both distributed in the Neotropical region.

### 3.2 Molecular Dating

The post‐burn‐in parameters of the BEAST analyses show ESS ≥ 200 for all relevant parameters. In the resulting dated phylogeny (see Figure 2 for the Coccinellini clade and Figure S2 for the complete tree), the MRCA of the Coccinellini tribe is estimated to have originated during the Paleocene, *c*. 65.25 Ma (95% HPD: 68.84–61.54 Ma). The median age of the MRCA of clade A (*Coccinella* group) is estimated at *c*. 54.9 Ma (95% HPD: 58.01–51.85 Ma), at the boundary of the Paleocene and Eocene. The three other major clades (clades B, C and D) all originated during the Eocene, with an estimated median age of *c*. 43.04 Ma (95% HPD: 45.85–40.16 Ma) for the clade B (*Cycloneda* group), an estimated median age of *c*. 41.38 Ma (95% HPD: 44.67–38.04 Ma) for the clade C (*Hippodamia* group), and an estimated median age of *c*. 37.15 Ma (95% HPD: 40.57–35.89 Ma) for the clade D (*Synonycha* group). Finally, a more recent earlier origin at the boundary of the Oligocene and Miocene is inferred for the clade E (*Pristonema* group), with an estimated median age of *c*. 22.2 Ma, albeit associated with a wide confidence interval (95% HPD: 44.11–5.27 Ma).

### 3.3 Historical Biogeography

The AICcω significantly supports (AICcω of 0.99) the DEC + j model as the best‐fitting model for the time‐stratified historical biogeography analyses. We also examined the results of the best‐fitting model without the +j parameter, because of raised conceptual and statistical flaws (Condamine et al. 2018; Ree and Sanmartín 2018; but see also Matzke 2022).

The Nearctic and East Palearctic regions are estimated to be the most likely ancestral area of origin for Coccinellini (see Figures 2 and S3 for the raw output from BioGeoBears). From there a vicariant event is inferred, leading to the colonization of the Neotropics by members of clade E on the one hand and a subsequent in situ diversification in the East Palearctic for the other lineages on the other hand. During the Eocene, from the East Palearctic, there was a range expansion (onto the West Palearctic and the Indomalaya), or the colonization of the Afrotropics and the Western hemisphere (for clade B) by other lineages. For the other major clades, members of clade A are thought to have originated in the East Palearctic, members of clade C in the East Palearctic and the West Palearctic, and members of clade D in the East Palearctic and Indomalaya. It was not until the Oligocene that the Australasian bioregion first began to be independently colonized by two lineages. During the Miocene, a more dynamic pattern is inferred, including reverse colonizations, northward dispersals and several colonizations of the Nearctic from the East Palearctic. The Pliocene and Pleistocene are inferred to be relatively uneventful save for additional colonization events of the Australasian region. The results of the best‐fitting analysis without the +j parameter (DIVA) give almost an identical biogeographic pattern, with the same inferred origin of the tribe in the Nearctic and East Palearctic regions and a similar pattern of range expansion and colonization (see Figure S4).

**FIGURE 3:**
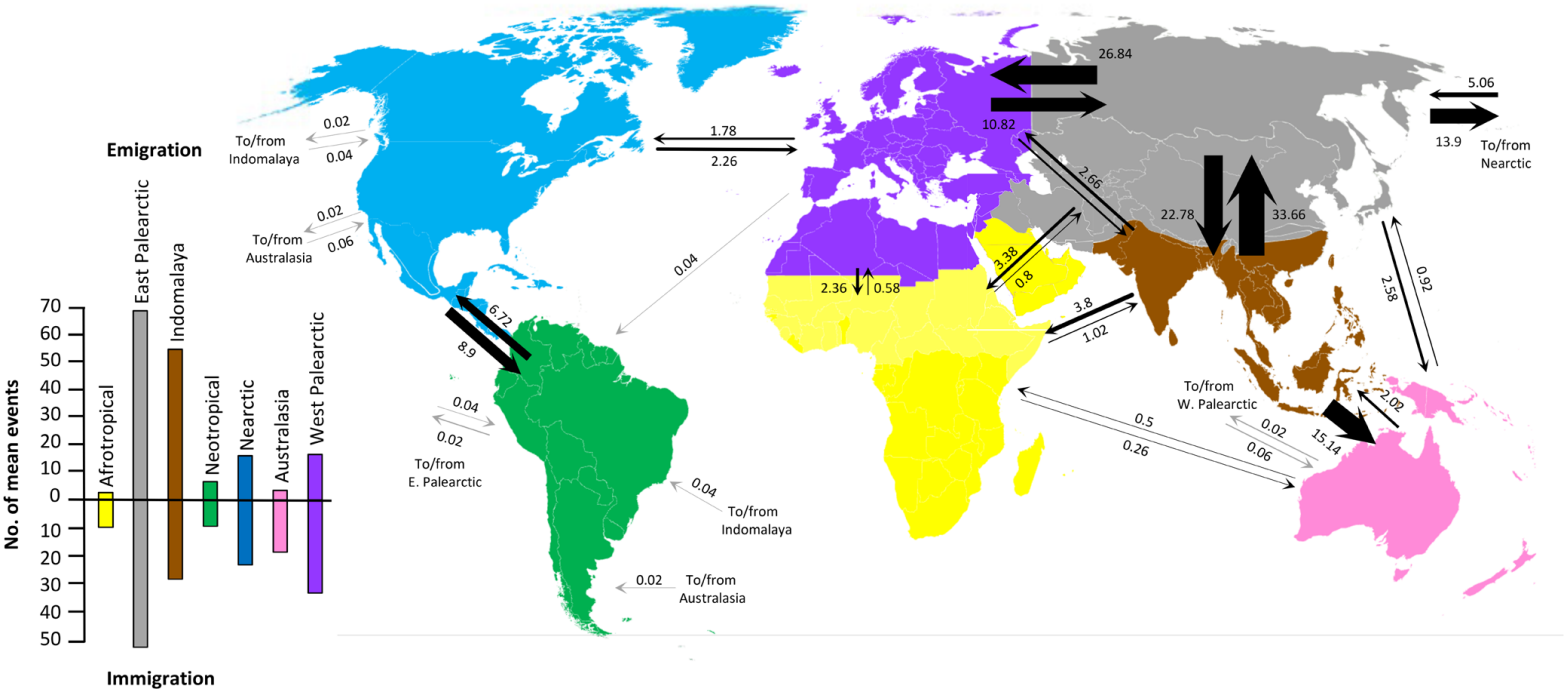
Results of the Bayesian stochastic mapping analyses, showing relative mean dispersal rates and immigration and emigration between bioregions.

The results of the BSM analyses based on the best‐fitting model (DEC + j; see Figure 3) underline the role of the East Palearctic as a major dispersal hub, with the highest emigration and immigration rates of all bioregions (69.52 and 51.28 mean events, respectively); they unequivocally support its role as a major colonization route toward the Nearctic. The Indomalaya also appears to be a key region, acting more as a sink with high emigration rates (55.34 mean events), almost exclusively toward the East Palearctic and Australasian regions. By contrast, dispersals in and out of the Afrotropical region are scarce (2.66 and 10.04 mean events, respectively). In the Western hemisphere, most faunal interchanges occurred between the Nearctic and Neotropical regions and they appear to be balanced.

### 3.4 Ancestral Character State Estimations

**FIGURE 4:**
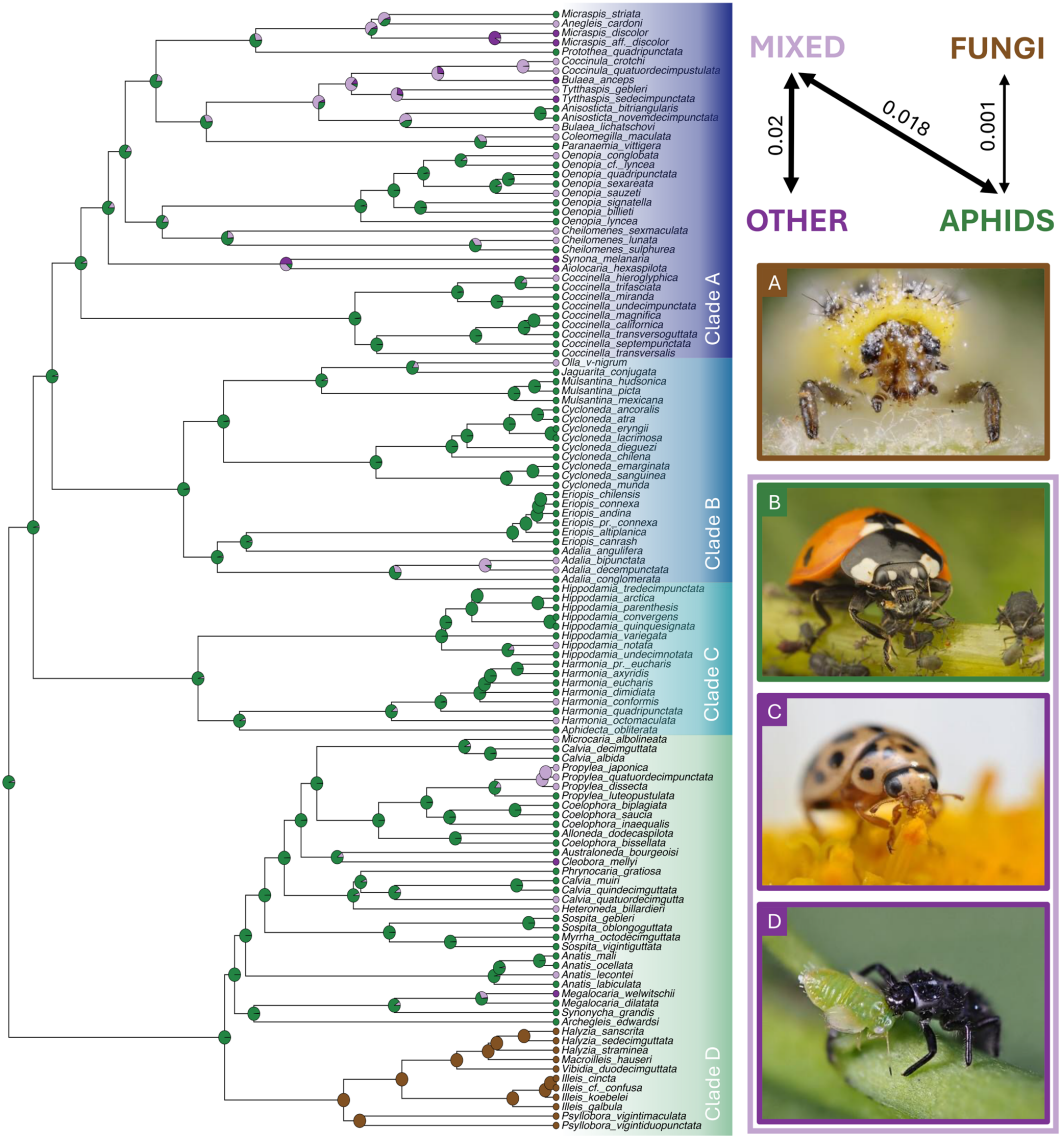
Ancestral character state estimation of diet evolution based on the best‐fitting model (SYM). The arrows between the four distinct diets provide information on transition rates and directionality. On the right, pictures of larval and adult ladybirds feeding on various food resources are displayed (pictures by M. Cole and G. San Martin).

The *fitMK* model comparisons support the SYM model as the best‐fitting model (AICω = 0.97) compared to the ARD and ER models (AICω = 0.03 and AICω = 0.00, respectively). The results of ASE analyses with the SYM model are presented in Figure 4, and they unequivocally support the hypothesis that the MRCA for Coccinellini was feeding on aphids. From there, most ancestral nodes (including those corresponding to the MRCA of clades A–D) are associated with a feeding specialization on aphids.

The best‐fitting model presents a clear directionality, as specialization on aphids can potentially lead to two distinct outcomes. The first is a shift toward mildew fungi, albeit with a low probability (transition rate of 0.001); the second has higher probabilities and first implies a broadening of the host repertoire (mixed diet; transition rate of 0.018), eventually leading (transition rate of 0.02) to the loss of aphid feeding and a specialization on another group of prey (heteropterans, psyllids, beetle or moth larvae). A dynamic pattern is inferred, as 17 independent shifts toward a mixed diet were recovered along the phylogeny, and these shifts sometimes lead to further shifts toward other specialized diets, outside of aphids. Only one direct shift from aphids to mildew fungi is inferred, corresponding to the specific diversification of a group within clade D, which in our sampling includes the genera *Halyzia, Illeis, Macroilleis, Psyllobora*, and *Vibidia*. Interestingly, two reverse colonizations on aphids are also recovered for the genus *Anisosticta* and *Micraspis striata* (Fabricius).

## 4 Discussion

### 4.1 Coccinellini Phylogenetic Relationships

The phylogenetic relationships between the obtained, distinct clades A‐E within Coccinellini are: (E, (D, (C, (B + A)))) (Figure 2). This is consistent with a previous study by Nattier et al. (2021) and Che et al. (2021), although contrary to Tomaszewska et al. (2021), where the relationship between these clades (A–D) was recovered as (A, (B, (C + D))). These differences are likely attributable to differences in terms of gene coverage and taxon sampling rather than methodological choice as all three studies relied on partitioned analyses under ML.

Within clade A, the predominantly Holarctic genus *Coccinella* is recovered sister to all other species with a high support (SH‐aLRT ≥ 80/uBV ≥ 95%), but it remains paraphyletic due to the highly supported (SH‐aLRT ≥ 80/uBV ≥ 95%) placement of the Afrotropical genus *Lioadalia*. This result was also found in previous studies with a similar high support (Escalona et al. 2017; Nattier et al. 2021; Tomaszewska et al. 2021) and is supported by morphological evidence (Iablokoff‐Khnzorian 1984). Similar to the results of Nattier et al. (2021) and Tomaszewska et al. (2021), *Aiolocaria* and *Synona* are found sister to all remaining species, with a high support (SH‐aLRT ≥ 80/uBV ≥ 95%). The Afrotropical genus *Cheilomenes* is recovered sister to the widespread *Oenopia* (SH‐aLRT ≥ 80/uBV ≥ 95%), in agreement with Nattier et al. (2021). The genus *Micraspis* is divided into highly supported two clades (SH‐aLRT ≥ 80/uBV ≥ 95%): one containing Asian and Australian species, and the other comprising Afrotropical species, along with the paraphyletic Afrotropical *Declivitata* and *Xanthadalia*, echoing the results of Tomaszewska et al. (2021). However, unlike Tomaszewska et al. (2021), we find the Indomalayan genus *Anegleis* sister to this Afrotropical clade, with moderate support (SH‐aLRT of 36/uBV of 95%). The highly supported (SH‐aLRT ≥ 80/uBV ≥ 95%) sister relationships of *Protothea* with this clade are consistent with previous studies. Except for the two *Bulaea* species and the widespread *Coleomegilla maculata* (De Geer), all remaining Holarctic species (*Aaages*, *Anisostica*, *Coccinula*, *Macronaemia*, *Paranaemia* and *Tytthaspis*) are gathered in the same clade, which is consistent with previous studies.

The clade B consists of Nearctic and/or Neotropical taxa, except for three *Adalia* species. This comprises three genus groups, where species restricted to Nearctic (e.g., *Cycloneda munda* (Say), *Mulsantina picta* (Randall)) are interspersed with species found only in Neotropical region (e.g., *Cycloneda emarginata* (Mulsant), *Paraneda pallidula* (Mulsant)). Contrary to Tomaszewska et al. (2021) and Nattier et al. (2021), we recover a weakly supported (SH‐aLRT of 12.4/uBV of 61%) Neotropical clade containing *Eriopis* and *Adalia angulifera* Mulsant, sister to a highly supported clade (SH‐aLRT ≥ 80/uBV ≥ 95%) grouping the remaining Palearctic *Adalia* species. All other relationships align with previous studies, including the highly supported (SH‐aLRT ≥ 80/uBV ≥ 95%) sister‐group relationship between *Cycloneda* and *Neocalvia*, and the clustering of *Jaguarita*, *Neda*, *Mulsantina*, *Olla* and *Paraneda* into a distinct and highly supported clade (SH‐aLRT ≥ 80/uBV ≥ 95%).

In clade C, we find the Western Palearctic genus *Aphidecta* sister to *Harmonia* (SH‐aLRT of 70.9/uBV of 99%), which occurs in all biogeographic regions except the Neotropics. Further, the *Aphidecta* + *Harmonia* clade is sister to the Holarctic genus *Hippodamia* with maximum support. This result aligns with Nattier et al. (2021), but differs from Escalona et al. (2017) and Tomaszewska et al. (2021), where *Aphidecta* is sister to *Harmonia* + *Hippodamia*, albeit with lower support (PP of 0.77/bootstrap value (BV) of 65% and BV of 74%, respectively).

The clade D comprises most of East Palearctic/Indomalayan and Australasian species. Among the non‐monophyletic genera, *Calvia* and *Coelophora* are particularly notable for their extreme polyphyly, being divided into multiple distinct groups, a situation already highlighted by Tomaszewska et al. (2021). This clade contains members of the *Halyzia* subgroup (*sensu* Tomaszewska et al. 2021), consisting of genera formerly classified in the tribe Halyziini (see Escalona et al. 2017).

The newly defined clade E consists in our sampling of representatives of two Neotropical genera (*Pristonema* and *Seladia*) that were previously classified in the tribe Discotomini (also transferred within Coccinellini in the *Discotoma* subgroup; Tomaszewska et al. 2021), which also included the Neotropical genera *Discotoma* Mulsant, *Euseladia* Crotch, and *Vodella* Mulsant. The phylogenetic analyses inferred that this clade is sister to the remaining representatives of Coccinellini (SH‐aLRT of 100 and uBV of 100%), echoing the results of Giorgi et al. (2009) who recovered *Pristonema* sister to the other sampled Coccinellini with maximum support. A more derived position of the representative of *Seladia* has also been found in other studies (Seago et al. 2011; Escalona et al. 2017; Tomaszewska et al. 2021), but with lower support. Here, we think that the inclusion of several members of the former Discotomini, combined with an expanded sampling of Coccinellini and molecular markers, has helped clarify the phylogenetic placement of members of this group.

### 4.2 Coccinellini Ancestral Area

Our median age estimate for the age of tribe (*c*. 65 Ma) is younger than the late Cretaceous origin inferred in the studies of Nattier et al. (2021) and Tomaszewska et al. (2021) (median ages of *c*. 84 Ma and *c*. 71 Ma, respectively). The hypothesis of a Paleocene origin for the tribe tends to be supported by the results of a recent genomic study on Coccinellidae (Huang et al. 2025), which estimates a younger age at the boundary of the Paleocene and Eocene (54 Ma; 95% HPD: 60–50 Ma). However, as representatives of clade E were not sampled in their study, their age estimate for the Coccinellini is therefore underestimated, further supporting the hypothesis of a likely Paleocene origin. The East Palearctic and Nearctic are unequivocally identified as the tribe's ancestral area (Figure 2), regardless of whether the +j parameter is taken into account. This result is consistent with the hypothesis of a Boreotropical origin during the Paleocene when there was a continuous warm‐temperate forest belt in the mid‐latitudes of the Northern hemisphere that persisted during the Early Eocene (Wolfe 1975; Manchester 1999; Wen 1999; Sanmartín et al. 2001; Tiffney and Manchester 2001). Such a Boreotropical origin has been evidenced in many lineages based on either fossil data or macroevolutionary analyses of extant taxa (e.g., see Archibald et al. 2014 for seed‐beetles; Durden and Rose 1978, Condamine et al. 2012, 2013, Allio et al. 2021 for swallowtails; and Zhang et al. 2022 for plants). Importantly, several studies also tend to support the hypothesis of a Northern Hemisphere origin in the boreotropics for several aphid groups (von Dohlen and Teulon 2003; Kim et al. 2011; Meseguer et al. 2015), which are the main prey of Coccinellini. The probable origin of aphids in the boreotropics is supported by fossil evidence and extant distribution patterns (von Dohlen and Teulon 2003), as well as by results of molecular dating and historical biogeography analyses (Kim et al. 2011; Meseguer et al. 2015). Following the progressive cooling occurring after the Early Eocene climatic optimum (EECO; 52–50 Ma; Zachos et al. 2001), the Boreotropical forest belt was gradually replaced by a mesophytic mixed forest, and many lineages began to disperse southward (Sanmartín et al. 2001).

### 4.3 Biogeographic Patterns Through Time

From the onset of their appearance *c*. 65 Ma, a first split is identified, leading to the southern dispersal in the Western hemisphere of one lineage (clade E) encompassing at least two genera (*Pristonema* and *Seladia*) that are today only distributed in the Neotropics (Figure 2). These two genera were previously classified in the tribe Discotomini, which is well defined morphologically by the shape and structure of the antennae (Tomaszewska et al. 2021); it is thus likely that clade E also includes the other three genera formerly classified in Discotomini, which are also only distributed in the Neotropics. There is considerable uncertainty about the timing of the colonization of the Neotropics by members of this clade, due to the very wide confidence interval of age estimates that were inferred and the non‐inclusion of all the genera formerly classified in Discotomini. The remaining lineages (clades A–D) apparently started diversifying in the East Palearctic, and there is no evidence of transcontinental dispersion between the Palearctic and the Nearctic through the De Geer or Thulean routes that were opened during the latest Cretaceous–early Cenozoic (Brikiatis 2014).

Major dispersal events are inferred during the Eocene (Figure 2). In clade C, the colonization of the West Palearctic is inferred prior to the appearance of the Turgai route in the Oligocene. Clades A and D both dispersed southward into the Indomalaya. In clade A, a lineage dispersed even further south into the Afrotropical region, likely through the Iranian route opened up at this time (Gheerbrant and Rage 2006). For clade B, we inferred a transcontinental dispersal between the Palearctic and the Nearctic consistent with the Beringian land bridge I route (70–20 Ma; Wen et al. 2016); colonization of the Neotropical region during the Eocene is also inferred for representatives of these lineages before the potential appearance of the GAARlandia land bridge *c*. 35 Ma. This could suggest the role played by the Antillean and Central American volcanic arc systems, which gave rise to non‐permanent islands that could have promoted biotic exchange between continents at this time (Iturralde‐Vinent 2006); such a pattern was inferred for example in a group of skipper butterflies (Toussaint et al. 2020).

In the Oligocene, two independent colonizations of the Australasian region are implied, predating the collision of the Australian plate with Southeast Asia (Figure 2). These events were likely favored by the emergence of a volcanic arc in present‐day Wallacea (Hall 2013), with ephemeral islands acting as stepping stones. Dispersal events from the East Palearctic to the West Palearctic are inferred, in line with the Turgai route. A transcontinental dispersal between the East Palearctic and the Nearctic, coinciding with the Beringian land bridge II (20–3 Ma; Wen et al. 2016), is also reconstructed.

Finally, from the Miocene onward, a highly dynamic pattern is inferred (Figure 2). In the Northern hemisphere, the Beringian land bridge II apparently played a central role, also implying that the associated Coccinellini lineages were cold‐adapted. Cold adaptation in Coccinellini is further illustrated by the dispersal events that have even been inferred from the time of the Beringian land bridge III (3–0 Ma; Wen et al. 2016), with arctic conditions associated with tundra vegetation. These dispersal events were probably made possible by adaptations to cold that are found in many Coccinellini genera, such as behavioral plasticity (aggregation behavior), plasticity in thermal physiology, phenotypic plasticity in color patterns (with larger spots at higher latitudes), and voltinism and dormancy (see the review of Sloggett 2021). In the Southern hemisphere, the colonization of the Afrotropics from Eurasia was likely favored by the collision of the African and Eurasian plates (similar dispersal events were inferred in other studies of insects; see for example Gustafson 2018; Toussaint et al. 2019, 2020; Tseng et al. 2022), which opened the so‐called *Gomphotherium* land bridge (Rögl 1998) through the Arabian Peninsula. Similarly, several dispersal events toward Australasia are potentially linked to the collision of the Australian plate and the development of Wallacea and the proto‐Papuan archipelago in the IAA (Lohman et al. 2011). In the Western hemisphere, the formation of the Panama Island Arc and the closure of Panama Isthmus are consistent with numerous biotic exchanges between the Nearctic and Neotropics that we inferred.

### 4.4 Emigration/Immigration and Major Dispersal Trends

The results of the BSM analyses provided overall trends based on dispersal events summed over the course of diversification of the tribe, and therefore they cannot reveal changes in rates over time. They underline the pivotal role of the East Palearctic, which is the region with the highest numbers of immigration and emigration mean events, ahead of the Indomalaya and far ahead of the West Palearctic and the remaining bioregions (Figure 3). There is a significant asymmetry between the Eastern and Western hemisphere, as most of the dispersal events occurred between the East Palearctic and the Nearctic, across the Bering strait; such a pattern matches what is predominantly observed in most plant and animal groups (Jiang et al. 2019). Almost no dispersal events were inferred between the West Palearctic and the Nearctic, likely because the De Geer and Thulean routes were only opened in the very beginning of the Cenozoic (Sanmartín et al. 2001; Brikiatis 2014), at a time when Coccinellini were likely not present in the West Palearctic. The Indomalaya also played an important role, acting as a sink with high numbers of emigration events, either toward the East Palearctic or Australasia. Very little exchange is inferred to and from the Afrotropics; although it could partially result from a sampling bias, we posit that it could be linked to the lack of diversity of aphids in the Afrotropics (Dixon et al. 1987). In the Western hemisphere, there is a quite dynamic and balanced pattern between the Nearctic and the Neotropical region. Finally, very few major long dispersal events are inferred, which suggests that Coccinellini might have relatively poor overseas dispersal abilities.

### 4.5 Diet Evolution

Ancestral state reconstructions inferred a highly‐supported ancestral diet association of Coccinellini with aphids (Figure 4), as already recovered in previous studies relying on a sparser taxon sampling (Escalona et al. 2017; Nattier et al. 2021). However, by having the former Discotomini (i.e., *Seladia* and *Pristonema*), whose diet is completely unknown, as a sister group to all remaining Coccinellini, the ancestral diet/food for the tribe is still open to question. The results of our historical biogeography analyses, combined with evidence from studies on aphids (von Dohlen and Teulon 2003; Kim et al. 2011; Meseguer et al. 2015), allow us to assume that both groups were codistributed in the Northern hemisphere at a time when a Boreotropical forest belt was present. This ancestral association with aphids supposedly involved a shift from Coccoidea (Giorgi et al. 2009; Seago et al. 2011; Escalona et al. 2017) and it is also associated with a change in mandible shape, from having an unidentate tip to a bifid one (Samways et al. 1997). Aphidophagy also implies a more opportunistic diet in relation with the life cycles of aphids (Escalona et al. 2017), which often make them an intermittent food resource. When aphids are scarce, aphidophagous species may integrate non‐prey foods in their diet such as fruit, honeydew, nectar or pollen (Lundgren 2009), and they can also target other non‐aphid prey, including ladybird eggs or larvae (even of their own species). This opportunistic behavior—where aphids are considered as essential prey that can be supplemented by alternative food resources (Hodek and Evans 2012)—has probably paved the way for the integration of new food sources in the host repertoire of Coccinellini. The results of ASE analyses suggest that a complete shift to a non‐aphid food resource first requires expansion of the host repertoire through a “mixed” diet (Figure 4). Interestingly, this pattern echoes the so‐called “oscillation hypothesis” first formulated for phytophagous insects, where recurrent oscillations between host expansions—the inclusion of new hosts into the diet—and specialization, have played an important role in insect diversification (Janz et al. 2006). In Coccinellini, we inferred transition to a mixed diet followed by either a specialization to a non‐aphid diet, or a reversal to a strict aphidophagous regime (for *Anisostica* and *Micraspis striata* ; Figure 4).

Shifts toward non‐aphid diet are generally not associated with substantial changes in mandible shape (Samways et al. 1997). However, there is an exception for fungi feeding that is associated with specific morphological key‐innovations such as the development of an additional serration along the incisor edge (*Halyzia* subgroup) or of a relatively stiff, comb‐like prostheca used to scoop spores and pollen (in former Tytthaspidini). Diet specialization on mildew fungi by members of *Halyzia* subgroup departs from the general “oscillation” model found for the other Coccinellini, as it is associated with a direct shift from an aphidophagous specialist diet without any prior expansion of the host‐repertoire. The cosmopolitan *Halyzia* subgroup (=Psylloborini, see Pakaluk et al. 1994) is comprised entirely of mycetophagous species (Gordon 1985), although some workers have also reported opportunistic aphidophagy or phytophagy (see the review of Sutherland and Parrella 2009). Sutherland and Parrella (2009) hypothesized that powdery mildew fungi are prevalent and abundant enough throughout the world for this group of beetles to maintain a specialized diet in a wide range of environments. This suggests that the transition from predation to mycophagy in this group—where the resource provides an easily accessible source of nutrients (Santamaria et al. 2023) – represents a significant ecological niche. This shift may also help explain the high diversity observed, as exemplified by the genus *Psyllobora*, which, with approximately 100 described species, is considered the most species‐rich genus within Coccinellini. Consistent with the former hypothesis, our age estimates suggest that members of *Halyzia* subgroup (genera *Eothea* Iablokoff‐Khnzorian, *Halyzia*, *Illeis*, *Macroilleis*, *Neohalyzia* Crotch, *Oxytella*, *Psyllobora* and *Vibidia*) were able to colonize all biogeographical regions in less than 20 million years, and that this spread may have been facilitated by the fact that powdery mildews were already globally distributed at that time (Takamatsu 2013).

## 5 Conclusion and Perspectives

This study yields new insights on the evolutionary history of ladybirds thanks to the integration of historical biogeography analyses and ancestral character state estimations on the most comprehensive sampling to date for the tribe. Although our sampling incorporates only one‐fifth of known Coccinellini species, the broad coverage that we achieved in terms of genera and biogeographic regions is expected to limit potential biases that are associated with uneven sampling when reconstructing ancestral areas (Turner et al. 2009) or trait evolution (Salisbury and Kim 2001). The joint inference of a boreotropical origin and an ancestral association with aphids is consistent with the results of studies postulating that they originate in the Northern hemisphere. The pattern inferred for the tribe suggests a dynamic biogeographic history under the influence of several major colonization routes, which is in agreement with major trends inferred for other insect groups. Diet evolution in Coccinellini reflects a history of opportunistic shifts often leading to an expansion of the host repertoire followed by secondary specialization, echoing the “oscillation model” proposed for phytophagous insects. The only deviation to this trend is the unique radiation of a mycetophagous lineage specializing on mildew fungi. Future studies on ladybird beetles will undoubtedly benefit from increased taxon sampling, but above all from the acquisition of novel knowledge about their life history. An interesting prospect in relation to the latter could also be the development of metabarcoding approaches to determine the gut content of ladybird larvae and adults. Recent advances in comparative genomics are also very promising, as underlined by the results of the study of Huang et al. (2025), who found that dietary shifts between distinct feeding regimes in ladybirds involve significant change in genomic architecture, especially in relation to the evolution of chemosensory, digestive, detoxifying, and immune genes.

## Author Contributions

**Romain Nattier:** conceptualization (equal), data curation (lead), formal analysis (equal), funding acquisition (lead), investigation (equal), methodology (equal), resources (lead), visualization (equal), writing – original draft (equal), writing – review and editing (equal). **Noémie M.‐C. Hévin:** conceptualization (supporting), data curation (supporting), formal analysis (equal), investigation (supporting), methodology (supporting), visualization (supporting), writing – original draft (supporting), writing – review and editing (equal). **Pascaline Chifflet‐Belle:** data curation (supporting), resources (supporting), writing – review and editing (supporting). **Jiajia Dong:** resources (supporting), writing – review and editing (supporting). **Hermes E. Escalona:** data curation (supporting), resources (supporting), writing – review and editing (supporting). **Julien M. Haran:** resources (supporting), writing – review and editing (supporting). **Jean‐Claude Streito:** resources (supporting), writing – review and editing (supporting). **Wioletta Tomaszewska:** resources (supporting), writing – review and editing (supporting). **Adam Slipiński:** data curation (supporting), resources (supporting), writing – review and editing (supporting). **Andreas Zwick:** data curation (supporting), resources (supporting), writing – review and editing (supporting). **Gael J. Kergoat:** conceptualization (equal), data curation (lead), formal analysis (equal), investigation (equal), methodology (equal), resources (supporting), visualization (equal), writing – original draft (equal), writing – review and editing (equal).

## Funding

The authors have nothing to report.

## Conflicts of Interest

The authors declare no conflicts of interest.

## Supporting information


**Figure S1:** Best‐fit ML tree resulting from the IQ‐TREE analysis with a perturbation strength of 1.0. Support values are provided on nodes (SH‐aLRT on the left and uBV on the right).

**Figure S2:** Dated phylogeny resulting from the BEAST analyses relying on a secondary calibration approach. Median age estimates are provided on nodes along with 95% HPD of ages represented by blue bars.

**Figure S3:** Raw graphical output from the BioGeoBears time‐stratified analysis relying on a DEC + j model.

**Figure S4:** Raw graphical output from the BioGeoBears time‐stratified analysis relying on a DIVA model.

**Table S1:** Primers used for PCR amplification.
**Table S2:** List of the 250 (including 206 Coccinellini) species included in the phylogenetic analyses along with GenBank accession numbers.
**Table S3:** Best‐fit partition schemes and models of molecular evolution inferred by ModelFinder.
**Table S4:**. Log‐likelihood scores of the best IQ‐TREE analyses with distinct perturbation strengths; the best analysis is highlighted in bold.
**Table S5:** Specimen distribution data for each sampled Coccinellini species. A, Afrotropical; E, East Palearctic; I, Indomalaya; N, Neotropical; R, Nearctic; U, Australasia; W, West Palearctic.
**Table S6:** Dispersal matrices for the time‐stratified biogeographic models. A, Afrotropical; E, East Palearctic; I, Indomalaya; N, Neotropical; R, Nearctic; U, Australasia; W, West Palearctic.
**Table S7:** Feeding habits of the Coccinellini species in our dataset and the categories used for the ancestral character state estimations.
**Table S8:** Detail of the vouchers and genbank accession of the 250 (including 206 Coccinellini) species included in the phylogenetic analyses.

## Data Availability

The data that support the findings of this study are openly available in Figshare at https://figshare.com/s/22482e2b7e82824f87b8. Newly generated sequences were registered and deposited in GenBank.
