# Supplementary figures and images for: Phylogenetic Analyses of True Ladybirds (Coleoptera: Coccinellidae: Coccinellini) Reveal Directionality in Diet Evolution and Support a Boreotropical Origin of the Tribe
Source: Ecol Evol. 2026 Mar 26;16(4):e73077. doi: 10.1002/ece3.73077 (PMC13107264; doi:10.1002/ece3.73077)

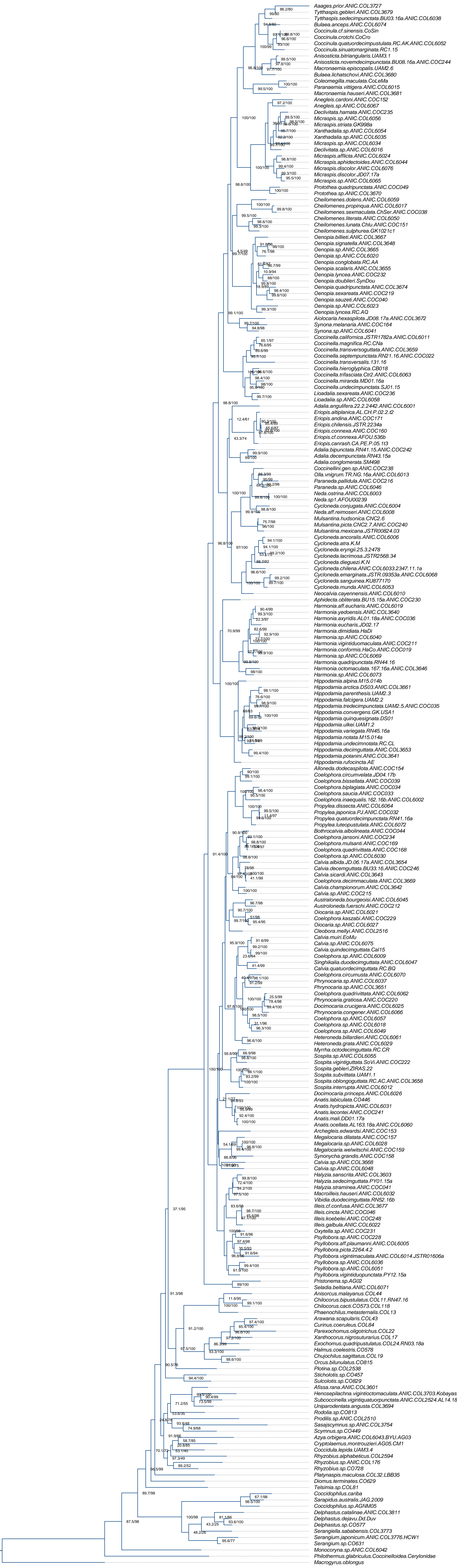

Supplement: Supplementary file 1 — Figure S1: Best‐fit ML tree resulting from the IQ‐TREE analysis with a perturbation strength of 1.0. Support values are provided on nodes (SH‐aLRT on the left and uBV on the right). [file ECE3-16-e73077-s005.pdf]

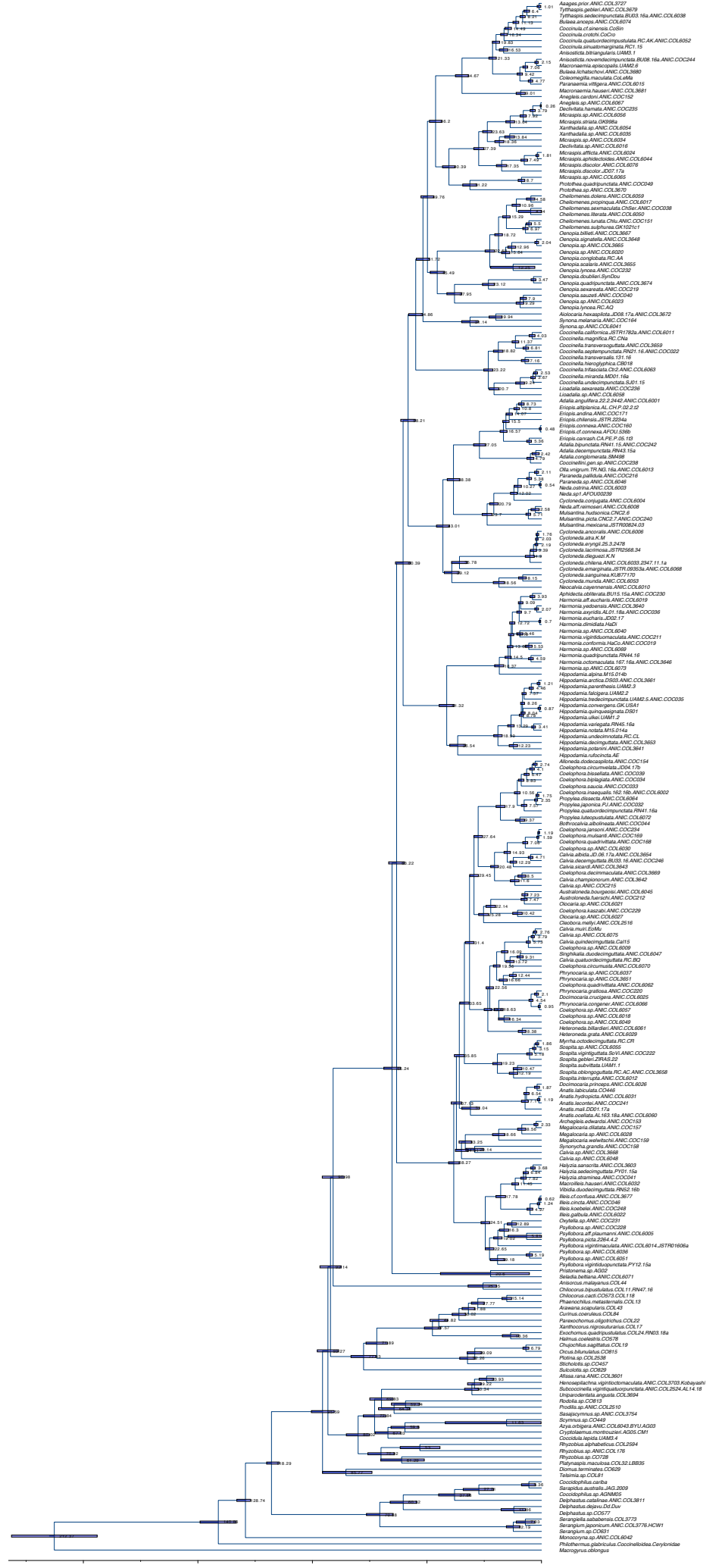

Supplement: Supplementary file 2 — Figure S2: Dated phylogeny resulting from the BEAST analyses relying on a secondary calibration approach. Median age estimates are provided on nodes along with 95% HPD of ages represented by blue bars. [file ECE3-16-e73077-s003.pdf]

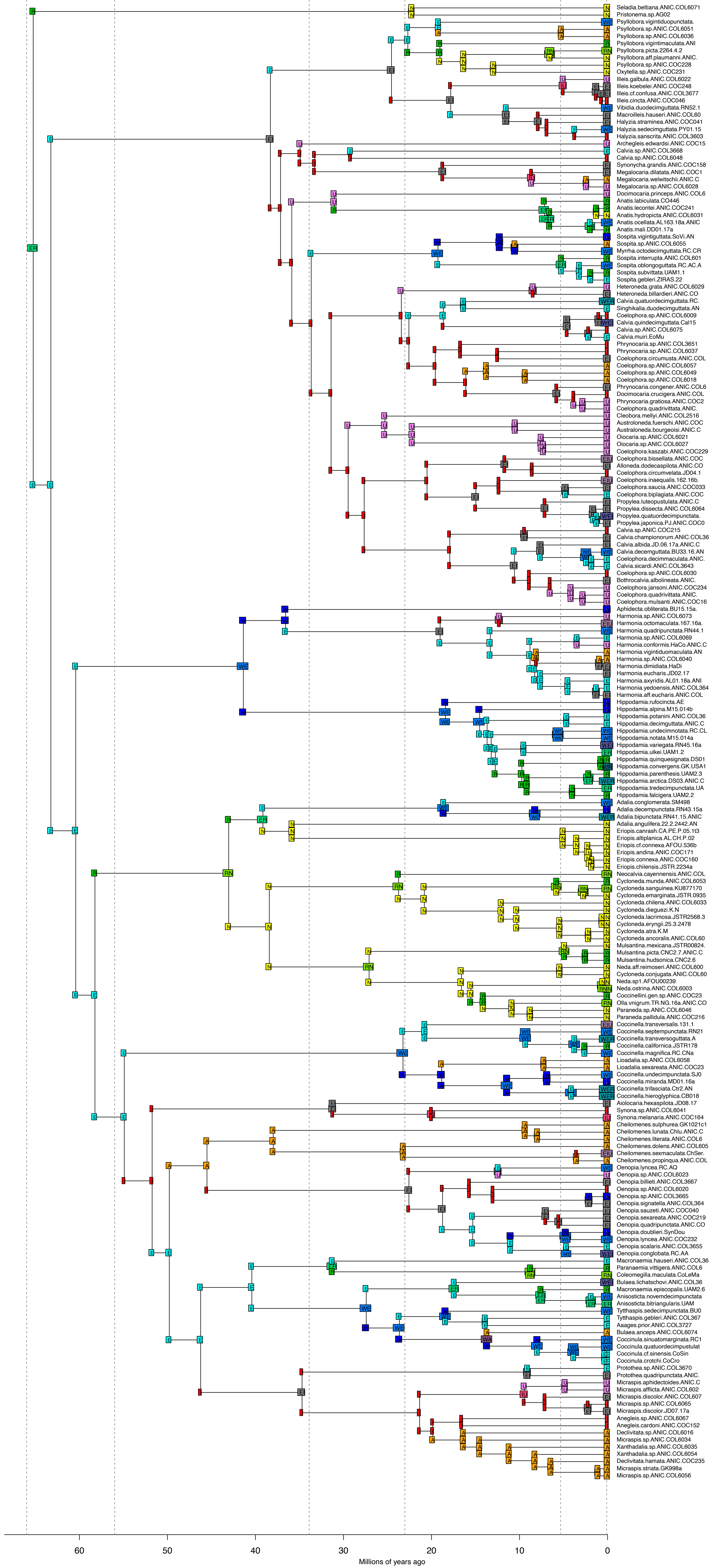

Supplement: Supplementary file 3 — Figure S3: Raw graphical output from the BioGeoBears time‐stratified analysis relying on a DEC + j model. [file ECE3-16-e73077-s002.pdf]

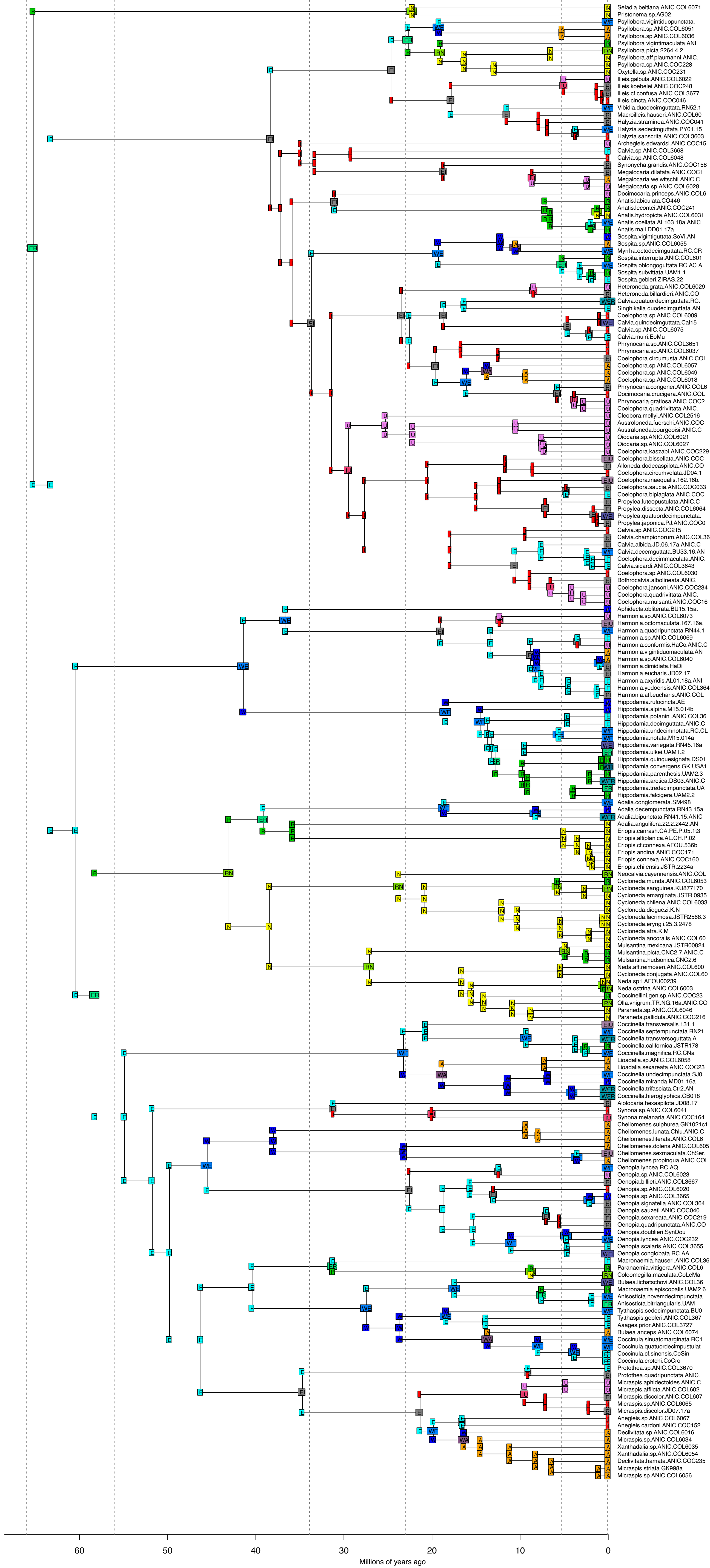

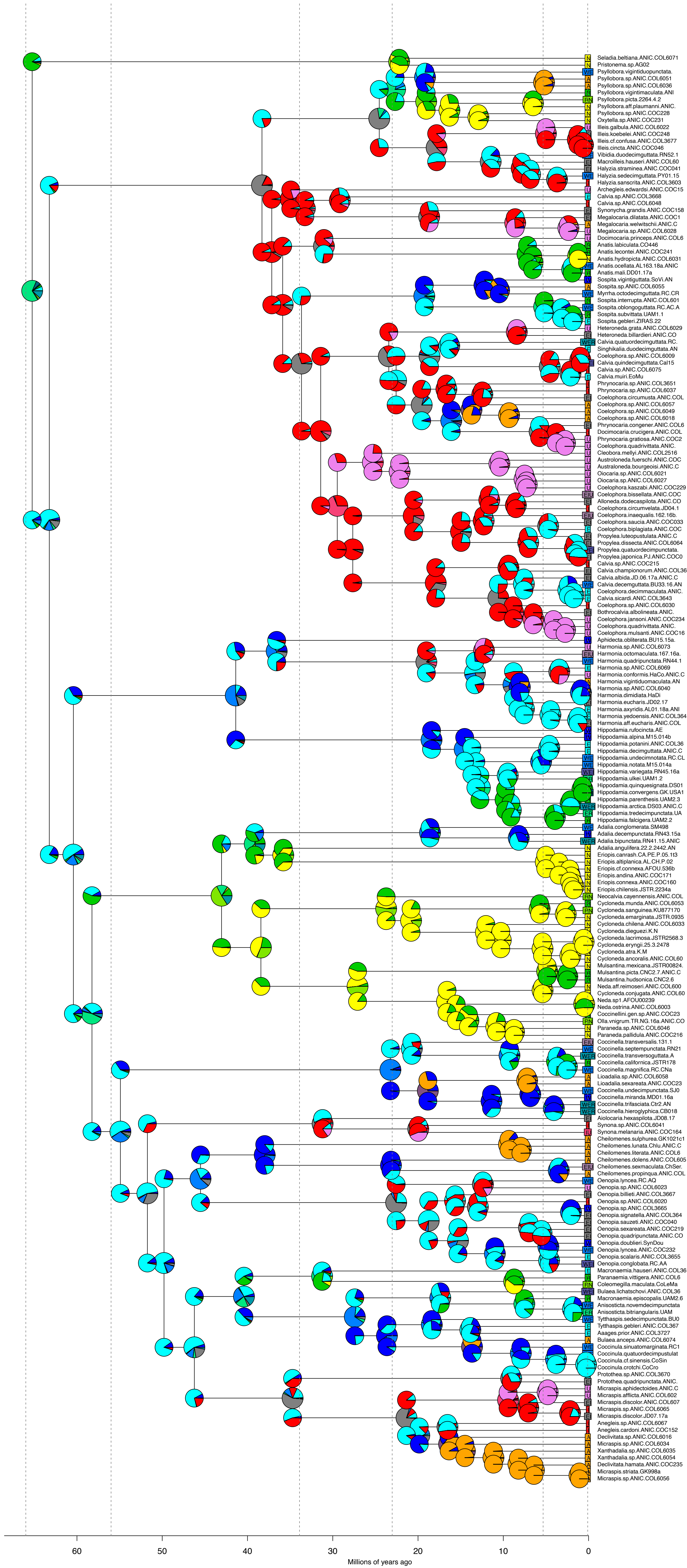

Supplement: Supplementary file 4 — Figure S4: Raw graphical output from the BioGeoBears time‐stratified analysis relying on a DIVA model. [file ECE3-16-e73077-s004.pdf]
